# Naturalization in a Context of Free Mobility: Evidence from Cross-National Data on Finnish Immigrants in Sweden

**DOI:** 10.1007/s10680-019-09530-3

**Published:** 2019-06-13

**Authors:** Jan Saarela, Kirk Scott

**Affiliations:** 1 Demography Unit, Åbo Akademi University, Strandgatan 2, 65100 Vaasa, Finland; 2 Centre for Economic Demography, School of Economics and Management, Lund University, P.O. Box 7083, 220 07 Lund, Sweden

**Keywords:** Naturalization, Cultural proximity, Cross-national data, Nordic countries, Return migration

## Abstract

Using unique longitudinal microdata linking administrative records from Sweden and Finland, we study how immigrant naturalization relates to cultural proximity. We analyze how Swedish citizenship acquisition depends on mother tongue by comparing Swedish-speaking and Finnish-speaking immigrants from Finland, who arrived in Sweden in 1988–2004, and contrast with other Nordic-born immigrants. We treat return migration and naturalization as two elements in the decision process of immigrants, being the first to estimate competing risks models for naturalization and return migration for the same study group of persons. The setting of free mobility in the Nordic countries, together with economic, political and social similarities, implies that the direct benefits of naturalization are modest and the same for all Nordic-born immigrants in Sweden. Thus, we assess naturalization in an analytical framework where many confounding factors are circumvented and in which the study groups have grown up in the similar institutional setting. Swedish-speaking Finns are found to have an approximately 30 percent higher standardized risk of naturalization than Finnish-speaking Finns, and a 2.5 times higher risk as compared to people from the other Nordic countries. We argue that these differentials reflect the degree to which the groups broadly differ in affinity with Sweden.

## Introduction

Immigrant naturalization, or citizenship acquisition, can in many respects be seen as a doorway to nationality-based privilege, and it is a subject of growing importance worldwide. Naturalization can be considered an observable sign of immigrants’ allegiance and commitment to the receiving country, and the extent to which immigrants are willing to become an integral part of the new society (Yang 1994). Understanding the naturalization process is consequently helpful in order to create policies that improve immigrant integration and socialization.

Since naturalization is a reflection of identity, cultural proximity between the home country and the host country is a key factor behind the decision to naturalize. The approach of previous empirical research concerned with the association between cultural similarity and naturalization has been to categorize immigrants into groups according to how “distant” the home country is from the host country. Generally, this refers to aspects such as similar traditions, a common history, and the use of the same language (see, e.g., Yang 1994; Dronkers and Vink 2012; Helgertz and Bevelander 2016). Isolating this proximity is difficult, however. The decision to naturalize takes place in an environment where a multitude of factors operate simultaneously, and the more heterogeneous the study population of immigrants is, the more difficult is it to isolate the influence of each contributing factor. In addition, an important decision that may compete with the naturalization decision is whether to eventually return migrate to the country of origin. As in the case of naturalization, the return migration decision is dynamic in nature and should preferably be analyzed within the same empirical framework.

This paper takes a novel approach to assessing the impact of cultural proximity on naturalization. We utilize a unique cross-country longitudinal dataset in a specific setting where we can assess the determinants of naturalization net of the obvious benefits of incorporation via the right to remain in a country and the access to nationality-based privilege. This is accomplished by examining the naturalization patterns of Nordic-born, and especially Finnish-born, immigrants in Sweden. The Nordic countries are economically, politically, and socially similar to one another and have been part of a free Nordic labor market since the 1950s. Citizens from any Nordic country have the right to settle and work in any other Nordic country without visas or other administrative procedures. As Nordic citizens receive the same rights as Swedes upon settlement, this provides us with an ability to examine naturalization in a setting where economic incentives are close to nonexistent, while the only direct advantages are the rights to vote and reside in specific restricted military areas. Thus, for a Nordic citizen, naturalization evidently reflects affinity with Sweden.

Finland is the focal country for this study since its population consists both of Finnish-speaking Finns as well as a minority of Swedish-speaking Finns. Almost all Swedish-speaking Finns in Finland live on the western and southern coastlines, and they amount to 5.5 percent of the country’s total population. The Finnish-speaking Finns constitute approximately 90 percent. Both groups are native and have equal constitutional rights. For many decades, emigration rates to Sweden have been higher among the Swedish speakers, and their return migration rates have been lower.

We exploit the fact that information regarding mother tongue is recorded in Finnish population registers and may be used to identify the linguistic background of Finns living in Sweden. As Swedish-speaking Finns identify more with Sweden than Finnish speakers do (Weber and Saarela 2019), this makes it reasonable to expect that they would be more likely to acquire citizenship in Sweden. However, there has been no study on this topic until now, because the two groups cannot be distinguished in the Swedish population registers. To do so, we have linked register data from Sweden to register data from Finland.

The case of Finns in Sweden allows us not only to disregard perceived economic incentives from the naturalization process, but also to analyze variation in cultural proximity within individuals from the same country of origin. Beyond the fact that the rights granted to Nordic citizens remove most of the economic incentives, an analysis of Finns by mother tongue implies that the study persons operate in the same context of reception, and come from the same native context. Danes and Norwegians are included as relevant comparison groups in the sense that their native languages are Scandinavian and thus highly similar to Swedish. Any variation in the naturalization risk between Swedish-speaking Finns, on the one hand, and Norwegians and Danes, on the other, is therefore not likely caused by linguistic differences. Since Swedish-speaking Finns identify themselves as ethno-linguistically Swedish, unlike Danes and Norwegians, any differences in naturalization rates can be claimed to be attributed to the latter group’s heightened cultural affinity with Sweden. This taxonomy allows us to speak more directly about the role of cultural proximity on naturalization in an analytical framework where many confounding factors are circumvented, and in which the groups have grown up in the same, or very similar, institutional setting.

The data consist of persons arriving in Sweden during the period 1988–2004 and contain socioeconomic, demographic, and labor market variables for each individual, with most being measured at the end of each calendar year. The primary focus of this study is the likelihood of becoming a Swedish citizen. Unlike previous research, we contrast the naturalization risk with the risk of return migration in the same study population. As is argued below, this is important from the point of view that there is strong selection in the return migration flow, and return migration and naturalization may be two competing elements in the decision process of immigrants. Thus, we obtain an unusually informative measure for how cultural proximity affects naturalization, and can make inference about the relation between return migration and naturalization within a competing risks framework.

Based on this setting, and what will be described next, we assume that, because of a closer perceived relationship with Sweden, Swedish-speaking Finns will have a higher rate of citizenship acquisition than Finnish-speaking Finns. To the extent that this differential is not based on language only, but on other aspects of identification as well, we should find that the Swedish-speaking Finns also naturalize at a higher rate than their other Nordic neighbors do. Unless selective return migration plays a strong role, Finnish-speaking Finns should naturalize at the lowest rate.

## Context and Previous Research

Since 1954, which was long before the pan-European dream of the European Union was realized, citizens of the five Nordic countries (Denmark, Finland, Iceland, Norway, and Sweden) have enjoyed rights of relocation and employment in all countries. No visas have been needed to settle or work, and the migration process has been similar to that of natives who migrate internally. This explicit right of residence minimizes the importance of naturalization on economic, social, and other outcomes of a more practical nature. As in the other Nordic countries, citizenship in Sweden is based on the *jus sanguinis* principle, that is, according to parents’ nationality (Clarke et al. 1998). Citizens from other Nordic countries can apply for Swedish citizenship after only two years of residence in Sweden, compared to the five years required for non-Nordic citizens. No language or naturalization tests, nor any income or employment requirement, exist. For Nordic citizens, Swedish citizenship acquisition by notification has also been possible, which means that the juridical naturalization procedure is simplified. Dual citizenship has been legally allowed in Sweden since July 2001, in Finland since June 2003, in Iceland since July 2003, while it is still not allowed in Denmark or Norway. The legislative change in Sweden was nevertheless simply a formal acknowledgment of an already existing liberal practice that allowed immigrants to possess dual nationalities (Spång 2007; Helgertz and Bevelander 2016).

Aggregate statistics for Sweden, referring to the proportion of the foreign-born population that is naturalized by country of birth, reveal a disparity between immigrants from Finland and those born in the other Nordic countries (Table 1). Danes, Norwegians, and Icelanders are among the least likely to be naturalized, while people born in Finland had naturalized at a level similar to migrants from refugee-sending countries. Except for the Icelanders, who are relatively few, the lion’s share of all naturalized people of Nordic descent had become Swedish citizens before the 1980s. Given the fact that the immigrants who come from Finland face no other benefits of Swedish citizenship than people born in the other Nordic countries, these numbers suggest the presence of some latent mechanisms and prompt the need to undertake longitudinal analyses.

**Table 1:** Foreign-born population in Sweden at the end of 2004 by continent or country of birth, percentage naturalized, and year of naturalization as % of all naturalized

Continent or country of birth	Total number	Naturalized	–1974	1975–1979	1980–1984	1985–1989	1990–1994	1995–1999	2000–2004
(A) Finland	186,589	63.8	42.3	20.6	15.0	9.1	6.7	2.7	3.4
(B) Denmark	41,663	45.4	55.0	14.2	11.7	6.7	4.9	3.8	3.7
(C) Norway	45,000	39.9	61.1	8.1	6.4	6.6	5.8	5.7	6.2
(D) Iceland	3851	18.0	14.3	6.2	9.8	9.0	11.1	12.4	37.1
(E) Other EU25 countries	183,943	59.4	39.9	12.7	9.9	12.6	8.9	5.5	10.6
(F) Other European countries	215,648	72.1	4.0	3.0	3.3	5.2	17.1	35.2	32.2
(G) Africa	65,249	61.3	4.5	3.7	5.6	7.7	20.4	27.1	31.0
(H USA and Canada	17,804	46.6	48.3	4.5	5.4	4.6	6.3	8.6	22.2
(I) Other American countries	64,199	69.5	5.8	5.6	11.5	16.6	23.4	18.3	18.9
(J) Asia	272,279	63.4	3.3	4.5	6.9	9.0	22.8	26.3	27.2
(K) Oceania	3517	32.7	24.9	8.4	8.1	6.3	9.7	13.3	29.3
(L) Unknown	520	25.4	13.6	0.8	1.5	3.0	18.2	37.9	34.1
Total	1,100,262	62.5	19.8	8.7	8.3	8.9	15.3	19.0	20.0

Other European countries include the former Soviet Union. The Swedish-born population amounted to 7,911,130 persons. The description refers to authors’ calculations based on Statistics Sweden (2005)

Many studies on the determinants of naturalization are on the North American context (Portes and Curtis 1987; Liang 1994; Yang 1994; Jones-Correa 2001; Bloemraad 2002, 2004; Chiswick and Miller 2009). Studies from Europe are of more recent date (Vink et al. 2013; Street 2013, 2014; Bevelander et al. 2015; Helgertz and Bevelander 2016). The decision to naturalize has been linked to a number of factors that operate at the individual, family, and contextual level, and which relate to both economic and noneconomic factors (Logan et al. 2012). Several specific conditions in the country of origin as well as in the host country, not to mention physical proximity between them, may therefore be important in promoting or discouraging naturalization (Yang 1994). In a setting where immigrants from a variety of countries are being studied, the decisions to return migrate and naturalize may depend on both the level of economic development and the degree of economic and political freedom in the country of origin (Chiswick and Miller 2009; Dronkers and Vink 2012). A common assumption is that an immigrant’s decision to naturalize is determined by the economic benefits available to citizens (Chiswick 1978). This may be in the form of earnings premiums paid to citizens, or access to employment that is closed due to discriminatory hiring behavior, both of which place non-citizens at a disadvantage (Becker 1971). Another potential advantage to naturalization lies in the possibility to access labor markets that would otherwise be restricted (Aleinikoff 2001), or that employers feel safer to employ citizens than non-citizens (Mazzolari 2009).

The roles of economic and noneconomic factors are nevertheless intertwined, since citizenship acquisition is likely the result of a multi-faceted process (Corluy et al. 2011). Together with demographic characteristics, the degree of cultural adaption to the host country is viewed as a key predictor of naturalization, because it is intimately related to successful integration (Yang 1994; Logan et al. 2012). Cultural proximity between the origin and the destination country is therefore expected to facilitate the process of naturalization. The study context here, with free mobility, full ability to return migrate, rights to employment and transfers, and no evident economic incentives for naturalization, provides a highly useful setting in order to understand how cultural proximity affects naturalization.

Naturalization may potentially also be interpreted as an outward expression of some underlying selection process (DeVoretz 2008). This would mean that the naturalization act itself is not the cause of greater economic success, but rather that those who naturalize would have been more successful even without the citizenship papers, and that immigrants who expect not to succeed may return migrate (Saarela and Rooth 2012; Saarela 2015). While some studies have found that naturalization is associated with subsequent improvements of the economic situation, primarily in terms of earnings (Bratsberg et al. 2002; DeVoretz and Pivnenko 2006), no such strong interrelations have been found in Sweden (Scott 2008; Helgertz et al. 2014). In some countries, but not in Sweden, access to government-supplied benefits requires citizenship, which would mean that individuals with tenuous ties to the labor market have greater incentives to naturalize (Borjas 2002; Euwals et al. 2010). In such circumstances, naturalization may be a sign of negative selection, rather than a stepping stone to economic success.

The most basic argument still is perhaps that naturalization signifies a long-term commitment to the country (Diehl and Blohm 2003). The decision itself may have no direct economic gains, but it is likely to result in, and be preceded by, actions with long-term benefits. This commitment may lead to, or be a reflection of, increased interaction with the native population, and thereby more natives in the migrant’s social network (Bloemraad 2002; Portes and Curtis 1987). Related factors are language acquisition and linguistic proximity (Chiswick and Miller 2014; Adserà and Pytliková 2015). Swedish-speaking Finns have for many decades had higher emigration than Finnish-speaking Finns, whereas their return migration rates had been lower (Saarela and Finnäs 2011). Income and employment levels of Swedish-speaking Finns in Sweden are at parity with those of native Swedes, while the labor market performance of Finnish-speaking immigrants is inferior (Rooth and Saarela 2007). Better labor market opportunities are not the sole explanation behind the different return migration rates, but it rather seems that affinity with Sweden plays an important role (Saarela and Scott 2017).

This notion relates to the fact that, besides its communicative function, language and ethnic identity are known to have a symbolic dimension (Edwards 1977; Fishman 1977; Gans 1979). Behind the linguistic facet, cultural norms and values distinguish Finnish-speaking and Swedish-speaking Finns (Saarela and Finnäs 2018). There are numerous identity markers related to group images, attitudes, and social structure, which separate the two groups and link Swedish-speaking Finns to Swedes (McRae 1999; Hedberg 2004). Migration to Sweden, in particular, is considered to be a mode of cultural expression, and to reflect a part of the identity of Swedish-speaking Finns (Hedberg and Kepsu 2003). Apart from any language barriers, differences in emigration patterns and return migration intensities are generally considered reflecting variation in commitment to and identification with Sweden (Weber and Saarela 2019). Although the division of Swedish-speaking and Finnish-speaking Finns is certainly not an automatic cause of cultural proximity to Sweden, it is in this context a reasonably good proxy, and one that goes beyond the standard approach of previous studies concerned with the antecedents of naturalization.

## Data and Methods

The data used were provided by Statistics Sweden (permission number 8547689/181453). They contain all documented moves into and out of Sweden in the period 1988–2005. Since we are interested in the naturalization rates of Nordic immigrants in Sweden, our focus is on people born in Finland, Denmark, Norway, or Iceland, who had a citizenship of any of these countries at the time of arrival in Sweden. For data secrecy reasons, Statistics Sweden collapsed Norwegians and Icelanders into one category at the time of data delivery, and we are thus forced to treat them as a single group. However, the Icelanders amount to barely eight percent of all persons in this group, so we can interpret the results as being driven by the Norwegians.

These population-based records include complete information about all individuals’ moves into and out of Sweden, even if they occurred prior to 1988. We have therefore restricted the data to ensure that individuals studied are first-time immigrants in Sweden on or after 1988. All individual characteristics, including citizenship, were measured at the end of each calendar year. We can thus prospectively assess both naturalization and return migration risks during the period 1989–2005 for all persons who lived in Sweden over the turn of a calendar year. These two events are treated as competing, while moves to a third country and deaths are right-censored.

For the immigrants who originate in Finland, there is linkage to information about the same persons when they resided in Finland. This part of the data was provided by Statistics Finland (permission number TK-52-215-11), and linked to the Swedish data via personal identification numbers. The population registers in Finland, unlike those in Sweden, record each citizen’s unique ethno-linguistic affiliation, usually referred to as mother tongue. The linkage consequently makes it possible to identify Finnish immigrants in Sweden according to whether they have Swedish or Finnish mother tongue, which would otherwise not be possible.

In total, the data include 31,961 immigrants from Finland, 27,057 immigrants from Denmark, and 44,599 immigrants from Norway and Iceland. Since Statistics Finland had a policy of not providing data on total populations, the Finnish part of the data constitutes a 77.5 percent random sample. Thus, our analytical data of the Finns include 7449 Swedish speakers and 18,071 Finnish speakers who can be linked to the data from Sweden. The number of persons who return migrate and naturalize, respectively, is 3604 and 523 for the Swedish-speaking immigrants from Finland, 11,910 and 750 for the Finnish-speaking immigrants from Finland, 15,906 and 558 for immigrants from Denmark, and 28,771 and 920 for immigrants from Norway and Iceland.

The Nordic countries have very detailed register data, and they share information with each other when people register for residence. Nordic citizens who move between the Nordic countries are required to register a move if they intend to stay for more than twelve months. There are, however, high incentives to register also shorter sojourns, since one cannot otherwise open a bank account, rent a flat, or receive income (Weber and Saarela 2019). Problems related to over-coverage of immigrants residing in Sweden are not a sensitive issue for migrants from the Nordic countries, and it is essentially a problem only for the few persons who leave Sweden for a non-Nordic country without deregistering (Monti et al. 2018).

To estimate the risk of return migration and naturalization as competing risks, we run discrete-time hazard regressions. People under risk of return migration are those who resided in Sweden at the beginning of each calendar year. People under risk of naturalization are those who resided in Sweden and did not have a Swedish citizenship at the beginning of each calendar year. Only 12.9 percent of all naturalized persons subsequently return migrate, which corresponds to 0.3 percent of all persons in the data.

In relation to the possibility of obtaining dual citizenship, we observed a threefold increase in the naturalization rate of Finnish immigrants in Sweden following the allowance of dual citizenship in Finland in 2003, compared to previous years. However, robustness checks show that inclusion of this period has virtually no influence on the estimates of interest (results available upon request). In the results reported, we have kept all years in the data, as that will extend the length of the follow-up and increase the data size.

Control variables consist of each person’s sex, age, time in Sweden, marital status, whether the person lived in a household with minor children, earnings, years of education, year of observation, and county of residence (*län*). For people originating in Finland, we can use also variables from their time in Finland that represent each person’s type of household, annual earnings, whether the person lived in owner-occupied housing, and region of residence (*landskap*). All these refer to the situation at the end of the calendar year prior to emigration from Finland. The variables and their distributions by country of origin are described in the next section. Separate analyses are undertaken for people aged 18+ years and for minors, respectively, considering that those in the latter group cannot themselves decide on migration or naturalization.

## Results

Nordic-born immigrants in Sweden were found to have notably higher return migration rates (cf. Monti 2018) and lower naturalization rates than immigrants in general, lying at levels that were roughly similar to migrants originating in Western Europe, North America, and Oceania (Table 2). There is, however, considerable variation within the Nordic group, and particularly between Swedish-speaking and Finnish-speaking immigrants from Finland. The Swedish speakers were not only less likely to return migrate than the Finnish speakers, but they were also more likely to naturalize. Approximately 54 percent of the Swedish-speaking Finns had returned to Finland after 15 years in Sweden, while 23 percent of those who still were living in Sweden by that time had Swedish citizenship. Corresponding numbers for the Finnish speakers were 71 percent and 17 percent, respectively. These unstandardized numbers also reveal that the proportion of return migrants among the Finnish speakers were roughly similar to that for people from the other Nordic countries, and they were slightly more likely to naturalize. The Swedish speakers, on the other hand, were more likely to remain in Sweden, and they naturalized to a greater extent than persons in the other Nordic-born groups.

**Table 2:** Migration and naturalization of the foreign-born population who immigrated to Sweden in 1988–2004, by origin of birth

Origin	Number	% Migrated after			% Naturalized after		
		5 years	10 years	15 years	5 years	10 years	15 years
(1) Finland, Swedish speaker	7449	30.6	48.7	53.7	3.7	13.2	23.0
(2) Finland, Finnish speaker	18,071	50.9	67.5	71.4	3.3	9.5	16.8
(3) Denmark	27,057	55.9	69.0	72.3	2.4	7.4	13.9
(4) Norway and Iceland	44,599	52.9	69.2	72.9	1.7	5.5	8.4
(5) Former Yugoslavia	114,222	3.5	6.9	14.3	45.3	83.9	83.6
(6) Western Europe	46,077	33.7	48.4	54.6	4.7	10.5	16.5
(7) Eastern Europe	66,365	7.3	12.9	16.0	26.3	59.7	76.6
(8) Southern Europe	28,348	9.6	17.1	22.5	17.0	54.7	71.9
(9) USA and Canada	15,734	44.3	58.5	62.0	5.0	9.7	20.2
(10) Central and South America	36,698	8.6	15.7	17.6	29.1	57.3	67.4
(11) Iran and Iraq	108,495	2.1	7.8	12.9	37.3	83.7	91.2
(12) Middle East	34,668	3.0	7.8	9.9	51.9	77.4	90.8
(13) Africa	57,480	5.4	12.9	18.4	32.3	76.7	85.7
(14) Asia	72,973	12.5	19.7	24.5	34.6	71.1	83.1
(15) Oceania	4422	42.6	61.6	73.7	4.4	10.6	22.4
(16) Unknown	129	5.0	16.4	20.0	32.9	63.0	75.0
Total	690,014	15.1	21.7	31.9	32.0	68.7	73.1

All persons studied had a non-Swedish citizenship at the time of immigration and lived in Sweden at the end of the immigration year

Calculations of the percentages migrated and naturalized are for persons with applicable time since immigration

The total number of persons born in Finland is 31,961. Swedish and Finnish speakers can be separated via linkage to data from Finland, which constitute a 77.5 percent random sample of the persons who migrated from Finland to Sweden. Approximately 3.3 percent of the people born in Finland who migrated to Sweden had a native language other than Finnish or Swedish

(5) Bosnia and Herzegovina, Croatia, Kosovo, Macedonia, Montenegro, Serbia, and Slovenia

(6) Austria, Belgium, France, Germany, Great Britain, Ireland, Liechtenstein, Luxembourg, Netherlands, and Switzerland

(7) Albania, Bulgaria, Czech Republic, Estonia, Hungary, Latvia, Lithuania, Poland, Romania, Russia, Slovakia, Slovenia, and other countries in the former Soviet Union

(8) Cyprus, Gibraltar, Greece, Italy, Malta, Portugal, San Marino, Spain, Vatican State, and Turkey

(12) Bahrain, Egypt, Gaza, Jordan, Kuwait, Lebanon, Oman, Palestine, Qatar, Saudi Arabia, South Yemen, Syria, West Bank, United Arab Emirates, and Yemen

(13) Excludes African countries in the Middle East

(14) Excludes Turkey, Iran, Iraq, and African countries in the Middle East

The groups also differed on individual characteristics (Tables 3 and 4). The Swedish-speaking immigrants from Finland were more likely to be younger, unmarried, to have immigrated more recently, to have higher earnings, to be higher educated, and to live in the Stockholm area. Finnish-speaking immigrants were more likely to live in Northern Sweden, while Danes to a higher extent lived in Southern Sweden, and Norwegians in regions close to the Norwegian border. A higher proportion of the immigrants from Finland were women, while men were overrepresented among those from the other Nordic countries. Since educational attainment is not automatically recorded upon immigration, there is missing information on years of education for many of the recent immigrants. However, since data from Finland can be used to assess educational attainment for the immigrants who originate in Finland, they do not suffer from this problem of missing information on years of education (cf. Saarela and Weber 2017). When comparing the Swedish-speaking and Finnish-speaking immigrants from Finland, we can additionally see that the former were more likely to had lived with their parents and in owner-occupied housing prior to emigration. A notably higher share of the Swedish speakers also came from Western Finland (Ostrobothnia and the Åland Islands), which is, together with Nyland in Southern Finland, the main settlement area of the Swedish speakers in Finland.

**Table 3:** Characteristics of Nordic-born immigrants aged 18+ years (%)

	Finland, Swedish speakers	Finland, Finnish speakers	Denmark	Norway and Iceland
Man	44.4	43.9	60.5	52.7
Immigrated before 1994	49.7	62.1	55.2	69.7
Age, 18–22 years	9.8	8.8	6.0	8.8
23–28	30.2	24.8	17.3	22.9
29–35	30.0	27.7	26.7	26.5
36–45	17.8	22.5	26.0	21.6
46–64	9.4	13.4	19.9	15.8
65+	2.9	2.8	4.0	4.4
Marital status, married	27.8	28.9	39.7	34.3
Not married	64.0	57.5	47.4	52.6
Previously married	8.2	13.6	12.9	13.1
Minor child in the household	27.6	31.1	33.6	38.0
Earnings in 2005 prices, no	18.4	24.6	35.1	28.1
< 50,000 SEK	13.6	16.0	11.3	14.7
50,000–99,900 SEK	8.6	10.9	7.9	10.0
100,000–199,900 SEK	21.7	22.9	18.4	22.5
200,000-299,900 SEK	22.4	16.5	14.9	16.3
300,000+ SEK	15.4	9.1	12.3	8.5
Years of education, < 10	18.0	28.5	14.0	17.0
10–11	13.9	18.9	12.0	16.0
12	19.7	19.0	10.7	13.1
13–14	18.0	13.5	8.3	9.0
15	17.4	12.2	7.7	6.3
16+	13.0	7.9	8.9	6.1
Unknown	–	–	38.3	32.4
County of residence, Stockholm	50.3	47.3	13.5	19.3
Norrbotten	2.0	12.8	0.7	2.4
Skane	4.4	4.6	43.5	7.8
Vastra Gotaland	5.7	7.5	8.9	23.5
Varmland	0.7	0.9	1.7	11.5
Other	36.9	26.9	31.7	35.5
*Type of household, single	25.1	33.9		
With parent(s)	50.9	36.7		
With partner and no children	11.2	12.3		
With partner and children	12.8	17.1		
*Earnings in 2005 prices, no	20.2	24.0		
< 5000 €	32.0	25.1		
5000–9900 €	13.7	14.3		
10,000–19,900 €	18.2	19.7		
20,000+ €	15.9	16.9		
*Owner-occupied housing	65.9	52.6		
*Region of residence, Nyland	25.0	28.3		
Ostrobothnia	41.3	3.6		
Aland Islands	19.9	0.5		
Lapland	0.0	14.6		
Other	13.8	53.0		
Number of individuals	6545	15,098	22,885	36,860

The description is based on the distribution of total time spent in Sweden

*Situation in Finland at end of calendar year prior to migration year. If education of Finnish-born immigrants is unknown in Swedish data, information from Finnish data has been used

**Table 4:** Characteristics of Nordic-born immigrants aged < 18 years (%)

	Finland, Swedish speakers	Finland, Finnish speakers	Denmark	Norway and Iceland
Boy	48.3	52.1	51.5	51.2
Immigrated before 1994	40.6	52.8	58.5	66.3
Age, < 7 years	25.2	25.4	30.7	28.9
7–12	43.9	44.3	40.9	41.6
13–17	30.9	30.3	28.4	29.5
County of residence, Stockholm	42.6	37.4	11.6	12.9
Norrbotten	3.4	15.5	1.3	2.3
Skane	5.9	4.3	35.1	11.5
Vastra Gotaland	4.3	7.6	8.3	19.4
Varmland	0.8	0.9	2.1	11.7
Other				
*Region of residence, Nyland	38.0	27.4		
Ostrobothnia	34.8	3.1		
Aland Islands	10.1	0.1		
Lapland	0.5	16.8		
Other				
Number of individuals	904	2973	4172	7739

The description is based on the distribution of total time spent in Sweden

*Situation in Finland at end of calendar year prior to migration year

To study whether compositional differences affect the between-group differences in migration and naturalization, we ran discrete-time hazard regressions of the relative risk to return migrate and to naturalize, respectively. Two models were estimated for each outcome of interest and age group. The first model used data on all Nordic migrants and adjusted for effects of all the control variables. The second model was restricted to data on Finnish-born persons only, but was able to exploit the cross-national character of our data, since we could then include characteristics referring to the time prior to emigration.

As related to Swedish-speaking immigrants from Finland, the unstandardized risk of return migration was 1.65 for Finnish speakers, 1.71 for Danes, and 1.58 for Norwegians/Icelanders. Compositional differences in characteristics across the groups contributed to only a modest part of the differences in return migration and in naturalization. With control variables included, the corresponding estimates were 1.66, 1.46, and 1.40, respectively (Table 5). The unstandardized risk ratios of naturalization were 0.69, 0.32, and 0.27, and the standardized ones 0.75, 0.42, and 0.32, respectively. Thus, the Finnish speakers naturalized at a significantly higher rate than the other Nordic migrants, while Swedish speakers naturalized at a rate that was 30 percent higher than that of the Finnish-speaking Finns. Finnish speakers were also the most likely to return migrate, while their Swedish-speaking countrymen had the clearly lowest return migration risk, or only about 0.6 that of Finnish speakers, and about 0.7 that of other Nordic citizens.

**Table 5:** Relative risks of return migration and naturalization (with 95% confidence intervals) for Nordic-born immigrants aged 18+ years

	Return migration		Naturalization	
	Model 1	Model 2	Model 1	Model 2
Origin				
(1) Finland, Swedish speaker	1	1	1	1
(2) Finland, Finnish speaker	1.66 (1.59–1.73)	1.57 (1.49-1.66)	0.75 (0.66–0.86)	0.77 (0.65–0.91)
(3) Denmark	1.46 (1.40–1.52)		0.42 (0.35–0.49)	
(4) Norway or Iceland	1.40 (1.34–1.46)		0.32 (0.28–0.37)	
Gender, Man	1	1	1	1
Woman	0.85 (0.83–0.86)	0.74 (0.72–0.77)	0.96 (0.88–1.05)	0.91 (0.80–1.04)
Time in Sweden, 1–4 years	1	1	1	1
5–6	0.67 (0.65–0.69)	0.67 (0.63–0.71)	7.30 (6.25–8.53)	9.33 (7.53–11.6)
7–8	0.54 (0.52–0.57)	0.56 (0.51–0.60)	6.92 (5.76–8.31)	8.88 (6.93–11.4)
9–10	0.46 (0.44–0.49)	0.49 (0.44–0.54)	8.17 (6.74–9.89)	9.21 (7.03–12.1)
11–12	0.35 (0.32–0.37)	0.41 (0.36–0.46)	8.32 (6.82–10.2)	9.41 (7.05–12.6)
13–14	0.24 (0.22–0.26)	0.25 (0.21–0.29)	7.45 (6.06–9.16)	10.5 (7.79–14.0)
15–17	0.23 (0.21–0.26)	0.22 (0.18–0.26)	9.11 (7.40–11.2)	13.2 (9.79–17.8)
Age, 18–22 years	1	1	1	1
23–28	0.89 (0.86–0.91)	0.81 (0.77–0.86)	0.41 (0.34–0.48)	0.39 (0.30–0.49)
29–35	0.77 (0.74–0.79)	0.67 (0.62–0.72)	0.26 (0.22–0.31)	0.23 (0.17–0.29)
36–45	0.67 (0.65–0.70)	0.53 (0.49–0.58)	0.27 (0.22–0.32)	0.23 (0.17–0.30)
46–64	0.51 (0.49–0.53)	0.43 (0.39–0.47)	0.20 (0.16–0.25)	0.17 (0.12–0.24)
65+	0.28 (0.26–0.30)	0.35 (0.30–0.41)	0.14 (0.09–0.21)	0.13 (0.07–0.22)
Marital status, married	1	1	1	1
Not married	1.00 (0.97–1.02)	0.90 (0.86–0.95)	0.70 (0.63–0.79)	0.70 (0.60–0.83)
Previously married	1.02 (0.98–1.05)	0.81 (0.76–0.88)	0.76 (0.65–0.90)	0.62 (0.48–0.79)
Minor child in the household, No	1	1	1	1
Yes	0.84 (0.82–0.86)	0.78 (0.74–0.83)	0.91 (0.82–1.02)	0.82 (0.70–0.96)
Earnings in 2005 prices, no	1	1	1	1
< 50,000 SEK	0.84 (0.82–0.86)	0.74 (0.70–0.78)	1.25 (1.07–1.47)	1.14 (0.91–1.41)
50,000–99,900 SEK	0.77 (0.75–0.80)	0.69 (0.65–0.73)	1.36 (1.14–1.63)	1.30 (1.01–1.67)
100,000–199,900 SEK	0.65 (0.64–0.67)	0.58 (0.55–0.61)	1.35 (1.16–1.57)	1.23 (0.99–1.52)
200,000–299,900 SEK	0.56 (0.54–0.58)	0.51 (0.48–0.54)	1.30 (1.11–1.52)	1.25 (1.00–1.56)
300,000+ SEK	0.77 (0.75–0.80)	0.67 (0.62–0.72)	1.18 (0.98–1.42)	1.14 (0.88–1.48)
Years of education, < 10	1	1	1	1
10–11	0.99 (0.96–1.02)	1.02 (0.96–1.07)	0.98 (0.85–1.13)	0.97 (0.78–1.20)
12	0.96 (0.92–0.99)	0.99 (0.94–1.04)	0.94 (0.82–1.08)	0.98 (0.80–1.20)
13–14	1.01 (0.98–1.05)	1.05 (0.99–1.12)	1.08 (0.92–1.26)	1.12 (0.89–1.40)
15	1.15 (1.10–1.20)	1.17 (1.09–1.25)	1.14 (0.97–1.35)	1.31 (1.05–1.64)
16+	1.21 (1.16–1.27)	1.14 (1.05–1.23)	1.17 (0.97–1.40)	1.30 (1.00–1.68)
Unknown	1.40 (1.36–1.44)		0.32 (0.25–0.43)	
*Type of household, single		1		1
With parent(s)		0.93(0.88–0.98)		0.94(0.78–1.14)
With partner and no children		1.19(1.13–1.26)		1.08(0.86–1.35)
With partner and children		1.42(1.33–1.51)		1.28(1.03–1.59)
*Earnings in 2005 prices, no		1		1
< 5000 €		1.16(1.10–1.22)		0.86(0.72–1.02)
5000–9900 €		1.21(1.14–1.29)		0.97(0.79–1.19)
10,000–19,900 €		1.17(1.10–1.24)		0.76(0.61–0.94)
20,000+ €		1.34(1.26–1.43)		0.77(0.61–0.98)
*Owner–occupied housing, No		1		1
Yes		1.13(1.08–1.17)		0.91(0.79–1.05)
Number of individuals (events)	81,388 (51,255)	21,643 (13,393)	81,388 (2055)	21,643 (1069)

County of residence (län) and year of observation are also included in Model 1, plus region of residence (landskap) in Model 2, but their estimates are not displayed here

*Situation in Finland at end of calendar year prior to migration year

Separate analyses for people who immigrated as children revealed that naturalization and return migration risks of Finnish speakers were on par with those of other Nordic citizens, whereas the Swedish-speaking child immigrants had a 50 percent higher naturalization risk and 35 percent lower risk of return migration (Table 6).

**Table 6:** Relative risks of return migration and naturalization (with 95% confidence intervals) for Nordic-born immigrants aged < 18 years

	Return migration		Naturalization	
	Model 1	Model 2	Model 1	Model 2
Origin				
(1) Finland, Swedish speaker	1	1	1	1
(2) Finland, Finnish speaker	1.57 (1.41–1.76)	1.57 (1.38–1.80)	0.67 (0.49–0.90)	0.57 (0.37–0.87)
(3) Denmark	1.57 (1.40–1.76)		0.67 (0.49–0.91)	
(4) Norway or Iceland	1.60 (1.43–1.78)		0.59 (0.45–0.79)	
Gender, Boy	1	1	1	1
Girl	1.01 (0.97–1.05)	1.03 (0.94–1.12)	0.89 (0.77–1.04)	0.84 (0.63–1.11)
Time in Sweden, 1–4 years	1	1	1	1
5–6	0.54 (0.50–0.58)	0.54 (0.46–0.63)	3.48 (2.79–4.33)	4.37 (2.87–6.65)
7–8	0.47 (0.42–0.52)	0.50 (0.41–0.61)	3.62 (2.74–4.77)	3.05 (1.77–5.26)
9–10	0.39 (0.34–0.44)	0.29 (0.21–0.40)	3.76 (2.78–5.09)	5.60 (3.29–9.51)
11–12	0.26 (0.22–0.32)	0.36 (0.25–0.52)	4.07 (2.87–5.78)	6.54 (3.60–11.9)
13–14	0.17 (0.12–0.23)	0.14 (0.07–0.30)	3.88 (2.51–6.00)	5.60 (2.26–11.8)
15–17	0.23 (0.15–0.35)	0.27 (0.11–0.66)	5.97 (3.48–10.2)	9.48 (3.75–24.0)
Age, < 7 years	1	1	1	1
7–12	0.86 (0.81–0.90)	0.76 (0.69–0.84)	0.62 (0.50–0.77)	1.26 (0.73–2.17)
13–17	0.89 (0.84–0.94)	0.78 (0.69–0.88)	0.53 (0.41–0.68)	1.63 (0.92–2.91)
Number of individuals (events)	15,788 (8936)	3877 (2121)	15,788 (696)	3877 (204)

County of residence (län) and year of observation are also included in Model 1, plus region of residence (landskap) in Model 2, but their estimates are not displayed here

It is important to stress that differentials in return migration risk by origin reveal a pattern that largely mirrors differentials in naturalization risk by origin. This pattern supports the view that return migration reflects unsuccessful integration (Saarela and Rooth 2012; Saarela 2015), while naturalization is an indicator of successful socialization (Yang 1994; Logan et al. 2012). Hence, even when having controlled for standard confounders, we found that Swedish-speaking Finns had an approximately 30 percent higher risk of naturalization than Finnish-speaking Finns, and a 2.5 times higher risk as compared to people from other Nordic countries. Since the benefits of naturalization are modest and similar for all Nordic immigrants, differences by origin are likely reflecting the degree to which the groups broadly differ in affinity with Sweden.

The estimated effects of the control variables were in line with much previous research (see, e.g., Yang 1994; Saarela and Scott 2017; Weber and Saarela 2019), and will therefore not be discussed at length. Time spent in the host country, as well as earnings in the host country, was negatively associated with the risk of return migration and positively related to the risk of naturalization. Women were less likely to return migrate than men, whereas the sex difference in naturalization was practically zero. Age decreased both the risk of return migration and that of naturalization. Married people were more likely to naturalize than non-married ones, whereas the association between marital status and return migration was less strong. A minor child in the household decreased the risk of both return migration and naturalization, whereas length of education had an increasing effect on the risk of both events. People who lived with their parents or as singles prior to migration from the home country were less likely than others to both return migrate and naturalize. Low earnings in the home country and having lived in owner-occupied housing were negatively associated with the return migration risk and positively associated with the naturalization risk.

## Conclusion

Persons who originate in any Nordic country have few, if any, economic incentives to naturalize in Sweden, and their naturalization rates are, therefore, notably lower than those of immigrants from other countries. The absence of strong incentives to naturalize has nevertheless been the key point of this paper, as it has helped to illustrate how naturalization relates to cultural proximity, within a context of free mobility by utilizing unique cross-national register data. The aim has been to examine differential rates of naturalization between Swedish-speaking and Finnish-speaking Finns, while contrasting these with other Nordic-born immigrants, and to consider that return migration and naturalization may be two competing events in the decision process of immigrants.

In line with expectations, the largest support for the argument that context of reception matters is provided by the case of the Swedish-speaking Finns. Within the group of Nordic-born persons, they have the lowest return migration risk and the highest risk of citizenship acquisition. The standardized naturalization risk for Swedish-speaking Finns is 30 percent higher than that of their Finnish-speaking compatriots.

To the extent that this differential is not solely based on language, but on other aspects of identification as well, we expected that the Swedish-speaking Finns should naturalize at a higher rate than their other Nordic neighbors do, and we do find that this risk is 2.5 times higher. Since there are no obvious incentives to naturalize, because Nordic citizens have equal rights in all countries, we find only modest effects of economic status on the naturalization risk. Given the linguistic similarity between Swedish, Danish, and Norwegian, we conclude that the substantial disparity between the Swedish-speaking Finns and other Nordic-born immigrants studied clearly suggests that cultural affinity and self-identification are crucial factors for immigrants’ decision to either return migrate or naturalize.

We argued also that, unless there is strong selective return migration, Finnish-speaking Finns should be expected to naturalize at the lowest rate, as they form the group of Nordic immigrants which is culturally and linguistically most distant from Sweden (Rooth and Saarela 2007). However, we found that they had a significantly higher naturalization risk than immigrants from the other Nordic countries. On face value, one may easily interpret this differential as a sign of negative selection in the inflow of immigrants, with Finnish speakers attaining citizenship to gain some advantage. However, such an interpretation is contradicted by the fact that naturalization grants no significant rights in terms of economic security in this context, and that the Finnish-speaking immigrants have the highest return migration risk of all Nordic-born immigrants. Additional regressions (results not shown) indicate that the influence of earnings and length of education on the naturalization risk is notably stronger for the Finnish-speaking Finns than for the other Nordic-born groups. The higher naturalization risk of Finnish-speaking Finns as compared to Danes and Norwegians is therefore likely the result of strong economic and social selection, indicating that the Finnish-speaking Finns who remain and eventually naturalize consist of a pool of positively selected citizens.

Our study setting can be considered unusually informative for making inference about the interrelation between cultural proximity and naturalization. It needs to be stressed, however, that the data used are at the individual level only, and we had no links to potential partners, children, or parents in the registers used. Previous studies have stressed the importance of the family situation, and particularly the origin-country characteristics of the partner, for naturalization (Street 2013, 2014). In Sweden, marriage to a foreign-born Swedish citizen increases the naturalization risk considerably, specifically when the spouse naturalizes during the same year (Helgertz and Bevelander 2016). Being married to a foreign-born Swedish citizen or to a native Swede has a larger effect on the naturalization risk for Nordic citizens than for other country-of-origin groups. However, it is known that less than one-third of all married Nordic-born immigrants belong to any of these two groups (Helgertz and Bevelander 2016), and of all people studied here, only one-third were married. The differential naturalization rate within the group of Nordic-born immigrants that we find cannot therefore be entirely attributed to the lack of partner information. This argument is supported also by the relatively modest effect sizes for how marital status and children affect naturalization, and how these variables improve the statistical fit of our models. Most of the other control variables are more important in this respect.

Another shortcoming of the data is that, since we have no link to parents of the study persons originating from Finland, we cannot separate persons with ethno-linguistically mixed background, that is, individuals with one Swedish-speaking parent and one Finnish-speaking parent. It is plausible that they, in terms of naturalization and return migration, lie somewhere in between persons with endogamous Swedish-speaking background and endogamous Finnish-speaking background. If that were the case, it would further corroborate the present arguments behind the link between cultural proximity and naturalization. In the years to come, we hope to gain access to cross-national register data that allow for analyses of links between children and their parents, and between household members.
